# Fateful Decisions of Where to Cut the Line: Pathology Associated with Aberrant 3′ End Processing and Transcription Termination

**DOI:** 10.1016/j.jmb.2024.168802

**Published:** 2025-01-01

**Authors:** Pawel Grzechnik, Hannah E Mischo

**Affiliations:** 1 Division of Molecular and Cellular Function, School of Biological Sciences, University of Manchester, United Kingdom; 2 Department of Infectious Diseases, School of Immunology and Microbial Sciences, King’s College London, United Kingdom

**Keywords:** 3′ end processing, transcription termination, read-through transcription, alternative polyadenylation, prremature transcription termination

## Abstract

Aberrant gene expression lies at the heart of many pathologies. This review will point out how 3′ end processing, the final mRNA-maturation step in the transcription cycle, is surprisingly prone to regulated as well as stochastic variations with a wide range of consequences. Whereas smaller variations contribute to the plasticity of gene expression, larger alternations to 3′ end processing and coupled transcription termination can lead to pathological consequences. These can be caused by the local mutation of one gene or affect larger numbers of genes systematically, if aspects of the mechanisms of 3′ end processing and transcription termination are altered.

## Highlights

• Poly(A)-signals (PAS) are modular, their usage is influenced by the availability of sequence elements and RNA binding proteins.
• In different sequence contexts, PAS sequences with varying usage-efficiencies have evolved.
• Throughout development, concentrations and affinities of PAS-binding proteins can change.
• PAS-dependent and -independent termination sites are both important to allow for fast gene regulation.
• Any change in these evolved equilibria can result in pathologies.

## Graphical abstract

**Figure:**
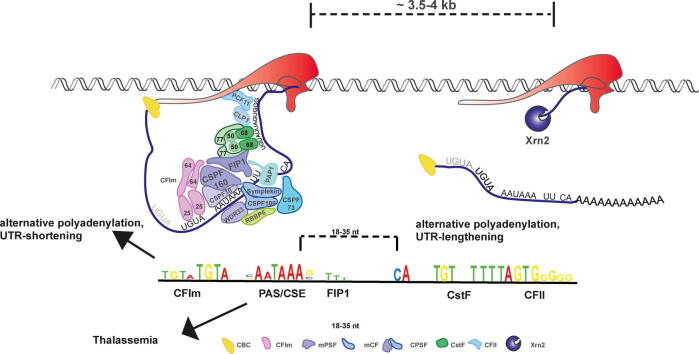

## Nothing is clear-cut

A cellular sensitivity to aberrant 3′ end processing can in part be explained by the complexity of this last co-transcriptional maturation step in the transcription cycle. 3′ end processing consists of two consecutive enzymatic steps that each affect various downstream processes.^1^ 3′ end processing requires multi-subunit protein complexes to bind to several RNA recognition elements that make up a poly(A) signal (PAS) as they emerge from the transcribing RNA polymerase II.^2^, ^3^, ^4^, ^5^ Coordinating 3′ end processing factor binding to the individual components of the PAS is not always perfect, as both the conservation and positioning of the individual recognition elements, but also the local availability of RNA-binding 3′ end processing factors can vary significantly.^6^, ^7^, ^8^ Pre-mRNA cleavage, the first enzymatic step, provides a 3′ end of the pre-mRNA, which is polyadenylated in the second enzymatic step. Polyadenylation is a prerequisite for mRNA nuclear export and subsequent translation.^9^, ^10^, ^11^ In turn, RNA cleavage also provides an RNA 5′ end which is required to remove the transcribing RNA polymerase II (RNAPII) from the DNA template.^12^, ^13^, ^14^ If RNAPII is not removed, its read-through activity can derange gene expression at further downstream loci and so explains why PAS mutations at one gene can affect expression of both the mutated but also that of a neighbouring gene.^15^, ^16^, ^17^ Local PAS mutations can have loss- or gain-of-function consequences, with a range of phenotypic consequences that can be selected for or against throughout evolution.^18^ In contrast, the concentration and activity of PAS-binding cleavage and polyadenylation (CPA) factors can vary, which will favour or exclude PAS usage at larger groups of genes.^19^, ^7^ CPA factor variations naturally occur in a tissue- and developmental-stage-dependent manner but can also be an indication of a pathology.^20^, ^21^, ^22^, ^23^ In the following, we will briefly delineate the modular nature of the 3′ end processing mechanism in healthy cells, before describing several selected monogenic and systemic consequences of aberrant 3′ end processing and transcription termination. As often with biological processes, many mechanistic details of both 3′ end processing and transcription termination were revealed through the study of mutations and pathologies. We refer the interested reader therefore to various comprehensive reviews that have focussed on complementary or similar aspects of mRNA 3′ end processing related pathologies in the past.^24^, ^25^, ^26^

### The sequence elements of the poly(A) signal (PAS)

**Figure 1:**
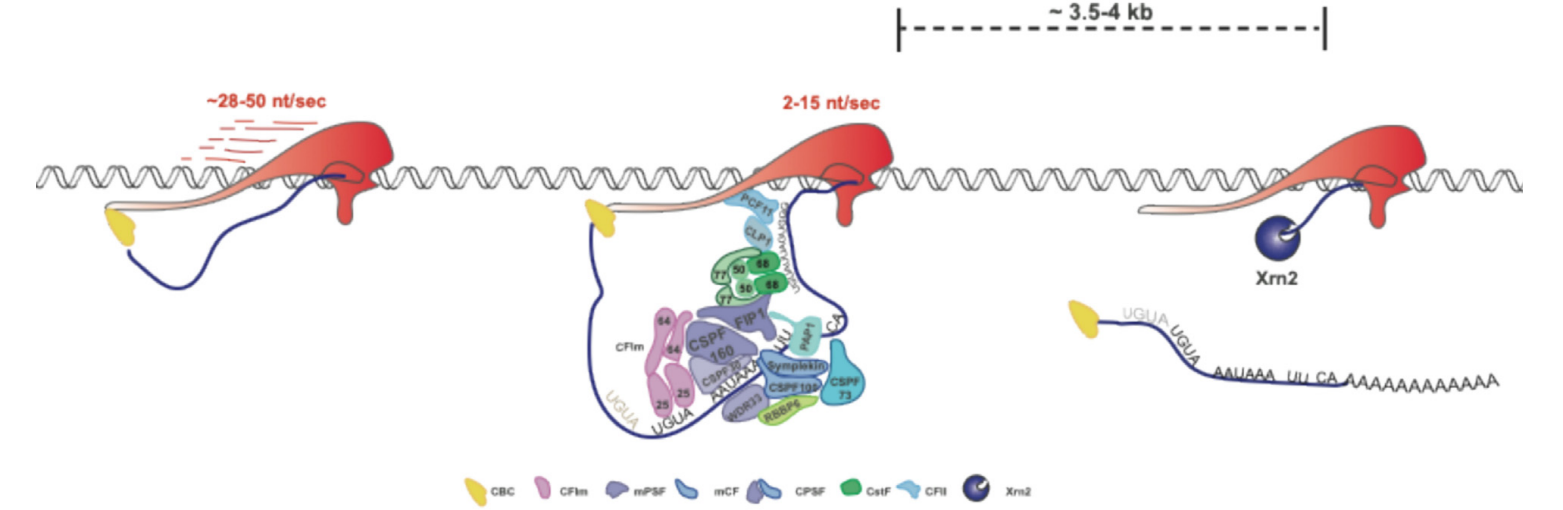
**Transcript 3′ end processing is prerequisite for transcription termination**. Upon transcribing a PAS, CPA factors assemble on the nascent pre-mRNA to form an active endonuclease. Consensus RNA binding motifs are indicated in the cartoon. CFIm binds as dimer – a second consensus sequence is indicated in grey letters. Pre-mRNA cleavage is followed by polyadenylation, which forms a pre-requisite for mRNA export from the nucleus. The nascent 5′ RNA end is engaged by Xrn2, which will degrade RNA up to the RNAPII body and so lead to destabilization of the elongation complex. Assembly of CPA factors and success of Xrn2 is also dependent on a decrease of transcription elongation speed mediated by complex rearrangements of elongation factors and influenced by post-translational modifications. Speed according to Cortazar et al.,^95^ termination zone according to Schwalb et al.,^98^ CBC: Cap binding complex (CBP20/80), CFIm; mammalian cleavage Factor I; mPSF; mammalian Polyadenylation specificity factor, mCF; mammalian Cleavage Factor, CPSF; cleavage and polyadenylation specificity factor, CstF, cleavage stimulating factor, CFII; cleavage factor II.

Co-transcriptional 3′ end processing removes mature mRNA from chromatin-bound RNAPII. This allows the mRNA to be handed over to the RNA export machinery and transcription termination to occur. 3′ end processing is initiated when a functional PAS emerges from the transcribing RNAPII and becomes available for CPA factors to assemble upon. Upon RNA binding, the endonuclease activity within CPA will be activated to cut nascent, PAS-containing RNA. Although assembly of the CPA factors on RNA is coupled to the transcription process, cutting of the pre-mRNA can occur with some delay, when all factors have bound to sequence elements up and downstream of the cleavage site.^27^, ^28^, ^29^ Once cut, the cleaved pre-mRNA is polyadenylated by Poly(A)-polymerase activities within the CPA complex and so prepared for export and translation in the cytoplasm (Figure 1).^30–32,^^11^

**Figure 2:**
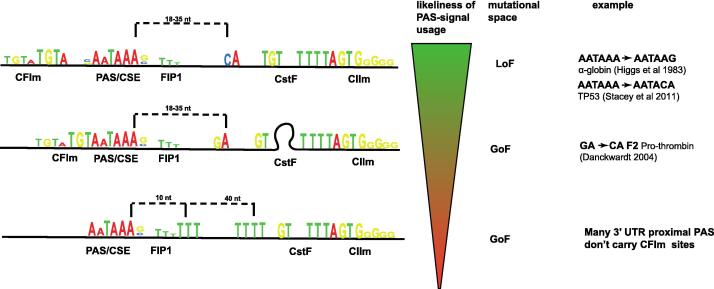
**Modular composition of PAS.** Modular composition of PAS creates a mutational space that determines the likelihood of PAS usage. Top: a strong PAS with optimal component spacing. Middle: suboptimal spacing is counteracted by secondary structure in the RNA. Suboptimal sequences within PAS elements are tolerated but lead to reduced cleavage activity (using alternative cleavage positions, here GA instead of CA). Bottom: lack of sequence elements (here the CA dinucleotide), will lead to a less accurate positioning of the cleavage position (sequence elements and position weight matrix adapted from Bogard et al.^6^ and Martin et al.^8^ represent a literal depiction of PWMs calculated in the respective papers. Cleavage position indicated as bracket from the CSE hexamer).

The central recognition sequence for 3′ end processing within the PAS is the consensus hexameric AAUAAA^2^ (Figure 2). AAUAAA hexamer, also referred to as the central sequence element (CSE), is recognised and bound by the mammalian cleavage and polyadenylation specificity factor (CPSF).^33^, ^34^ Approximately 30–60 nt downstream of CSE, two copies of the heterotrimeric Cleavage stimulating Factor (CstF) will bind a U/GU-rich site.^35^, ^36^, ^37^, ^38^ G-rich sequences further downstream are bound by PCF11, as part of the heterodimeric mammalian cleavage factor II (CFII/CFIIm).^39^ Binding of all these factors is required to stimulate CPSF to cleave pre-mRNA 10–27 nucleotides (nt) downstream of the CSE, in most eukaryotic organisms either at a CA or UA dinucleotide.^40^, ^41^, ^42^, ^43^, ^44^, ^45^, ^46^

Upstream stimulatory elements (USE) can also aid recruitment of CPSF, when CstF recognition sequences are missing, when CSE has a reduced consensus or in genomic surroundings where high efficiency processing and transcription termination are required; such as in several virally encoded pre-mRNA or gene-dense regions, e.g., complement factor C2 or in the lamin B gene.^47^, ^48^, ^49^, ^50^ Often USEs contain a UGUA consensus sequence upstream of the CSE.^51^ Binding of mammalian Cleavage Factor I (CF Im) to this sequence will stimulate cleavage efficiency but is not essential for the cleavage reaction.^52^, ^53^, ^43^, ^44^

Of these multiple elements, the hexamer AAUAAA or CSE is the best conserved and tolerates few variations.^54^ However*, in vivo* about 40% of all genes carry single nucleotide AWUAAA variations (W = A/T) that seem to be tolerated if the other PAS sequence elements are well conserved and optimally spaced from one another.^40^, ^18^, ^6^^,55–59^ Optimal distances between the different elements of the PAS will enable interactions between proteins of the different subcomplexes whilst they assemble on the RNA to form the active endonuclease. Extensive, but weak secondary structures close to CSE and cleavage sites can bridge and juxtapose sequence elements that would be too far apart in a linear sequence arrangement.^55^, ^50^

It was early noted that IgM, one of first sequenced genes, can be polyadenylated at two different PASs. A full-length IgM is expressed from an mRNA isoform polyadenylated at a PAS in the 3′ untranslated region (3′UTR) downstream of the last exon. In contrast, a second mRNA isoform is expressed from a PAS located within the last exon, producing an IgM protein isoform with a different and shorter C-terminus.^60^, ^61^ Analysis of expressed sequence tags (ESTs) and later poly(A)-tail-directed sequencing found that around 50% of genes can use two or more alternative PASs to end the same messenger RNA.^62^, ^56^, ^57^, ^40^, ^58^, ^63^ These alternative PASs can be located as in the case of IgM in an exon and so affect the amino-acid sequence of the encoded protein (denoted coding-region (cr) alternative polyadenylation (APA)). Alternatively, additional PASs can be found in 3′UTRs and so affect 3′UTR length, called 3′UTR APA.^25^, ^64^ As 3′UTRs are often the sites of micro (mi)RNA- and RBP-mediated posttranscriptional control, varying the 3′UTR length of an mRNA can be an efficient way to remove regulatory sites and so alter protein levels without changing transcription rates and RNA concentration of a given gene.^63^

Newer estimates indicate that the average human gene contains as many as five PASs.^59^ A large proportion of these (81%) reside in promoter-proximal introns and are rarely used. If these intronic poly(A) signals (IpA) were employed, they would give rise to truncated proteins and so their use is largely suppressed in nonproliferating cells.^65^, ^66^, ^67^, ^68^, ^69^, ^70^ Whilst most mRNA use a preferred PAS, usage of PAS can change during cellular differentiation or pathology.^71^, ^63^, ^72^ However, importantly, PAS usage does not uniformly change at all genes, but instead depends on context-specific binding of RNA binding proteins (RBP) to PAS accessory sequence elements found in specific groups of genes.^63^, ^23^, ^53^

Given this flexibility and modular design of the PAS, a wide spectrum of cleavage and polyadenylation efficiencies is observed and it has generally proven difficult to predict what effect PAS mutations have on individual genes.^18^, ^59^ However, mutations in a gene’s hexameric CSE more likely abrogate its usage, which results in read-through transcripts that are not exported and so lead to depletion of that mRNA from the translatable mRNA pool.^15^, ^73^, ^32^, ^30^, ^31^ In contrast, mutations in the PAS-surrounding elements do not necessarily preclude PAS usage. Mutations in the PAS-accessory elements influence CPA factor binding and endonuclease activation and so alter the likelihood of PAS usage.^6^, ^18^, ^40^ Anecdotally, a PAS is described as weak, when its elements are sub-optimally spaced or conserved, and is defined as strong when its elements are well-conserved and so used with high efficiency. As discussed below a change in PAS usage can profoundly affect the protein pool of a cell and so may lead to pathological consequences.

### The components of the CPA complex

Apart from effecting a spectrum of cleavage and polyadenylation efficiencies, modular PAS also provide an insurance policy to the cell, as RNA cleavage only occurs at a PAS on which all the enzymatic activities required for cleavage and polyadenylation have been assembled. Thus, CPSF, initially purified as one complex most likely exists in cells as two subcomplexes, mammalian polyadenylation stimulatory factor (mPSF) and mammalian Cleavage Factor (mCF).^35^, ^34^, ^74^, ^75^ mPSF, is formed by a scaffold of CPSF160, two RNA binding proteins CPSF30 and WDRR33 that directly contact the PAS, and FIP1.^33^ Successful PAS recognition by CPSF30 and WDR33 is communicated by FIP1, which is positioned on RNA towards the cleavage site (with a preference for U), but also makes contacts with CSTF2 (aka CSTF64) and PAP to aid tethering of these two to the assembled cleavage complex.^76^, ^77^, ^78^, ^79^, ^80^ The heterotrimeric CstF consists of CSTF77 (CstF of 77 kDa, aka CSTF3), CSTF64 (aka CSTF2) or CSTF64τ and CSTF50. CstF will bind to GU-rich sequences immediately downstream of the cleavage site and is also required to stimulate pre-mRNA 3′ end processing.^43^, ^37^, ^35^ Recruitment of the active endonuclease requires an individual factor RBBP6 to mediate contact between mPSF and the endonuclease CPSF73 within mCF.^43^ In analogy to the very conserved yeast system, this binding likely induces a conformational change in the endonuclease CPSF73 (mCF) and so licences it for cutting.^44^, ^43^, ^81^, ^82^, ^83^ Finally, CFII, consisting of CLP1 and PCF11, has a weak binding preference to G-rich sequences that in strong PAS are found 3′ of the CstF binding site.^39^

PCF11, similar to its yeast counterpart, is a prerequisite for the cleavage reaction, but has also two other important functions^43^, ^44^; PCF11 will bind to the transcribing polymerase and so couple 3′ end processing to ongoing transcription.^84^, ^85^, ^86^, ^87^, ^88^, ^5^ Secondly CFII (PCF11 and CLP1) will stimulate transcription termination by aiding the recruitment of the 5′–3′ exonuclease XRN2, which is required to disengage RNAPII from the DNA template.^89^, ^90^, ^85^, ^91^, ^14^ XRN2, also dubbed the molecular torpedo, will degrade the nascent RNA still attached to RNAPII, which has lost most of its elongation factors and is significantly slowed down downstream of the PAS.^92^, ^93^, ^94^, ^95^, ^96^, ^97^, ^12^, ^13^, ^14^ Yet, whilst XRN2 degrades its way down the nascent RNA, RNAPII still traverses on average about 4 kb before it is eventually removed from the DNA template.^98^

This last step in the transcription cycle is controlled through an intricate protein interaction network that ensures directionality of the enzymatic processes and controlled specificity. In addition to these core polyadenylation factors, several RNA-binding proteins can bind sequences near the poly(A) signal to either promote or inhibit PAS usage. Such a regulatory function has been strongly implicated for CFIm, which comprises two serine-arginine-rich (SR) protein components CFIm68 or CFIm59 and CFIM25.^53^, ^52^ CFIm, when bound to the UGUA-consensus, can recruit FIP1 and so act as an RNA cleavage enhancer.^53^, ^43^, ^99^, ^100^ Therefore, PAS that contain a CFIm binding site, will preferentially be used if CFIm is expressed.^53^, ^101^ Similarly, when CPA-complexes contain more or less PCF11, FIP1 or PAP1, this will affect PAS preference.^43^, ^44^, ^102^, ^7^, ^103^

Thus, usage of a PAS depends on three main factors: (1) Sequence positioning and conservation. (2) Availability of core CPA factors. (3) Presence of auxiliary factors that can be either stimulatory or inhibitory. Machine learning and network analysis are currently aiding a mechanistic understanding on the interdependency of the individual sequence elements with a focus on auxiliary elements that can act cooperatively or antagonistically.^18^, ^6^, ^75^, ^104^, ^59^

A further layer of control is conveyed by posttranslational modifications (PTM) that act on both the transcribing RNAPII, as well as CPA factors. Most notably, spatially controlled phosphorylation of the C-terminal domain (CTD) of RNAPII coordinates association of elongation and co-transcriptional processing factors with the transcribing RNAPII and so is an essential regulator of all aspects of transcription.^105^, ^106^, ^107^ Similarly, the enzymatic activity of several CPA factors or their ability to bind to RNA is controlled through phosphorylation^104^, ^108^, ^109^, ^110^, ^111^, ^112^ or ubiquitin-mediated processes.^113^, ^114^, ^115^, ^116^, ^109^, ^117^ The importance of PTMs for 3′ end processing factors is emphasised by the organisation of the budding yeast Cleavage and Polyadenylation factor (CPF); about half of CPF stably associates with a phosphatase module which combines several phosphatase activities that are required to coordinate 3′ end processing and transcription termination in budding yeast.^118^, ^119^, ^108^, ^120^ In human and plant genes, some of these yeast interactions and functionalities appear conserved. Thus, mCF has been shown to bind the phosphatase Ssu72, even though no stably associated phosphatase complex has been identified thus far.^44^, ^121^, ^95^

As with all other essential pre-mRNA processing steps, the core factors involved in 3′ end processing are essential, and their complete deletion precludes survival.^122^ However, hypo- or hypermorph mutations (especially in model systems, such as budding yeast) have been instrumental in understanding how fine-tuned the process of 3′ end processing in a healthy cell is that leaves little room for error.^4^, ^84^

## PAS variations and cis mutations causing read-through transcription

### Loss-of-function mutations

Due to their high abundance and importance in erythrocytes, the globin family of genes has from the infancy of molecular biology been under intense scrutiny. Globin genes have not only proven a fruitful model to understand gene expression control and associated pathologies. But their study has also pioneered gene therapy methodology to ameliorate monogenic diseases in man. Thus, β-globin was among the first two genes to be sequenced from their poly(A) tail and the first cDNA to be cloned.^123^, ^2^ 3′ end sequencing revealed the conserved AAUAAA of the poly(A) sequence as key signal required for polyadenylation and export.^2^, ^124^, ^73^ Hemoglobinophathies were demonstrated to correlate with less globin production due to either faulty or reduced mRNA levels.^125^ Globins were also the first genes through which it was shown that RNA 3′ end processing and transcription termination are two separate,^126^ but coupled mechanisms.^17^ Thalassemia is a genetic disease associated with severe anaemia, first observed in Mediterranean countries with a wide spectrum of penetrance ranging from a mild to a life threatening condition with low life expectancy.^127^, ^128^ The latter anaemias are generally transfusion-dependent and aside from the reduced life quality for the patient, present a major burden to health care systems with multimillion investments per patient.^129^

Tetrameric haemoglobin contains dimers of α-like and β-like globin monomers which switch in composition during erythroid cell development. During the first year of life, foetal haemoglobin HbF, consisting of α_2_γ_2_ is replaced by adult haemoglobin in which α-chains are combined with either β (HbA, α_2_b_2_) or δ chains (HbA_2_, α_2_δ_2_). α- and β-thalassemias, resulting from reduced α- or β-globin expression, therefore have an onset within the first year.^127^, ^129^

α- or β-globin mutations are broadly separated into two classes; Often mutations result in premature stop codons, which will generate a substrate for nonsense mediated decay but more rarely mutations occur outside the coding region in the 3′UTR surrounding the PAS.^130^, ^131^ These PAS mutations in the β-globin gene were the first identified PAS-mutations that showed that the human poly(A) sequence, just as had previously been shown for the SV40 PAS is required for polyadenylation and RNA export (see Figure 2).^124^, ^130^ Several different PAS mutations have been described in α- and β-globin genes that contribute to the wide variety of genetic backgrounds in thalassemia that in some patients can occur in combination.^132^, ^133^, ^134^, ^130^, ^135^

Early therapies transfusing patients with HLA donor matched haematopoietic stem cells (HPSC) to prevent regular blood transfusions, were hampered by donor-availability and high rates of graft rejection.^129^ In an attempt to modify the donor’s own cells, two new therapies use CRISPR-Cas9 or Zn-finger nucleases.^136^, ^137^ Both therapies aim to increase foetal γ-globin expression and so bypass the need for the adult β-globin. In both therapies, γ-globin expression is increased by interfering with expression of the repressive transcription factor BCL11A. Exagamaglogene autotemcel (Exa-cel) uses CRISPR-Cas9 to cut at a GATA1 transcription factor binding site within the BCL11A enhancer. If repaired by non-homologous end joining, this usually results in deletion of the GATA1 binding site and so abrogates BCL11A expression in a tissue specific manner. To prevent off-target effects of the CRISPR-Cas9 nuclease, edited cells are routinely checked by sequencing for additionally acquired mutations. Although this therapy also requires patients to undergo strong chemotherapy prior to infusion, the first treated patients remained transfusion independent and had no clinical complications for over 15 months.^138^, ^136^ Exa-cel has been approved for treatment of transfusion-dependent thalassemias at the end of 2023.

Although strictly speaking neither of these therapies repairs the original mutation, newer developments are on their way that may enable on-site genome correction. Thus, a recent publication tested the feasibility of using prime-editing, another variation of the CRISPR-Cas9 technology, in which Cas9 is fused to a reverse transcriptase and an extended guide RNA that will serve as genome-sequence correcting template.^139^, ^140^ The success of these therapies opens an important health policy debate for the coming years to decide on how these gene therapies will be priced, and their use regulated.

### Gain-of-function mutations

Scrutiny of available genome sequences compared to all possible PAS mutations shows that there appears to be a selection against loss of function mutations.^18^ Yet, mutations that reduce (hypomorph) or increase (gain of function or hypermorph) the efficiency of 3′ end processing are more frequent and appear enriched in some disease-spectrums.^18^ As one example, a suboptimal single allele-specific A to C substitution in the TP53 PAS from AAUAAA to AAUACA greatly reduces polyadenylation efficiency and expression of the tumour suppressor p53.^141^ Consequently, this TP53 PAS polymorphism is associated with multiple cancers.^142^, ^141^ One of the earliest described gain-of-function mutations is the cause for haemophilia, a disease in which blood clotting proceeds too fast. In haemophilia, Thrombin (F2) is expressed at too high levels, as its usual suboptimal cleavage site within the prothrombin gene is mutated from CG to CA and so leads to more protein production (see Figure 2).^143^

With the growth of genomic databases linked to clinical phenotypes, (e.g., UK Biobank, ClinVar, Genomics England, gnomAD), more and more single nucleotide polymorphisms (SNPs) that are predicted to affect 3′ end processing are being discovered.^25^, ^26^, ^59^ However, as evidence of the complex relationship between proper 3′ end processing for balanced gene expression, 48% of 1723 SNPs identified in ClinVAR sequences, were predicted to result in increased PAS strength while 52% may cause decreased strength. A case by case mechanistic analysis will be required to better understand these genotype-phenotype relationships.^59^

## Systemic read-through or genome-wide disruption of PAS recognition

The examples above have shown how defined mutations of individual PAS can affect gene expression locally. However globally, about 34% of all human genes suffer from inefficient 3′ end processing at their PAS.^144^ As transcription termination requires cleavage at the PAS, RNAPII will continue transcribing beyond these genes and so produce read-through transcripts. Read-through transcription at any given PAS is generally relative to the overall transcript levels of the upstream gene, but there is no correlation between the overall level of transcription and the likelihood of read-through at a given gene.^144^ This observation reinforces the notion that the sequence context of a PAS directly determines its effectiveness.^6^, ^18^, ^59^ In some genomic contexts, strong PAS signals have been selected for, whereas in other contexts the evolutionary pressure for fast 3′ end processing appears lower.^144^

**Figure 3:**
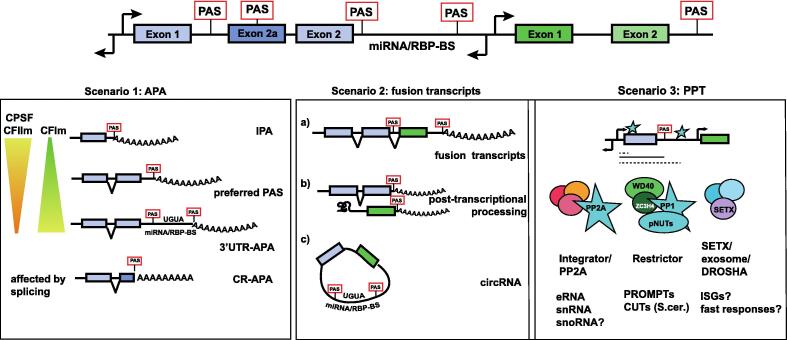
**Consequences of systemic changes to PAS usage.** Scenario 1 describes different outcomes of APA that depend on local concentrations of CPA components; CPSF/CFII depletion more likely results in global distal PAS usage, CFIm depletion more likely results in proximal PAS usage; IPA: intronic polyadenylation. CR-APA; coding region APA. Scenario 2: failure to use PASs can lead to fusion transcripts or favour splicing over 3′ end processing possibly resulting in circular RNA formation. Scenario 3: PPT (promoter-proximal termination), also referred to as PTT (premature transcription termination) in the literature. In addition to CPA-mediated termination at least two (integrator and Restrictor), but possibly 3 (including SETX) other mechanisms have evolved that can lead to premature or promoter proximal transcription termination.

The fate of these read-through transcripts depends on both, how the transcript is severed from the transcribing RNAPII and how RNAPII is eventually removed from the DNA template. In turn, both these events are influenced by the downstream genomic context and can in principle result in three different outcomes for the RNA, described as scenarios in the following sections (Figure 3).

Scenario 1: RNAPII reads through a PAS and the read-through transcript is 3′ processed at a downstream PAS and creates a new mRNA isoform with a longer 3′UTR.

Scenario 2: RNAPII reads through a PAS, the transcript is processed at a downstream PAS which may belong to a downstream gene and so create a bi-cistronic mRNA.

Scenario 3: RNAPII is terminated at non-canonical or non-PAS termination sites. These non-PAS-dependent termination mechanisms are collectively called non-coding termination mechanisms, as they result in non-polyadenylated RNA that is usually not translated. In budding yeast, RNAPII reading through PAS at the end of genes can also be terminated by a non-coding (nc)RNA termination mechanism. As the budding yeast ncRNA termination pathway is coupled to nuclear RNA degradation, read-through transcripts are usually immediately degraded.^145^, ^146^, ^147^, ^148^, ^11^ In mammals, several separate mechanisms supporting non-PAS RNAPII-termination may result in different RNA fates. However, it is not clear, if these can act downstream of regular PAS.^147^, ^97^

However, it is becoming increasingly clear that fast gene expression control requires crosstalk between PAS-directed and non-PAS-directed transcription termination. Thus, genes that require fast induction are often constitutively expressed, but their transcription constantly terminated close to promoters through non-PAS dependent mechanisms. Upsetting such promoter proximal transcription termination can prevent cells from adapting to new environmental situations.^149^, ^150^

### Scenario 1: Alternative polyadenylation (choose wisely)

The first genome-wide attempts to define PAS for each mRNA, indicated global differences in PAS usage between highly proliferating cancer, activated immune and developing cells, in contrast to differentiated, and senescent cells.^71^, ^151^, ^72^, ^102^, ^152^, ^153^ During cellular differentiation, PAS closest to the last exon are progressively read-through, giving rise to longer 3′UTRs.^72^, ^154^ Conversely, up to 90% of mRNA of various cancer cell lines were shown to preferentially use proximal PAS, leading to shorter 3′UTRs.^153^, ^71^, ^155^ These global trends are supported by sequence context of individual PAS that will support binding of 3′ end processing promoting or inhibiting factors.

#### CPA stimulators and enhancers

Most prominently, UGUA, the sequence bound by CFIm, is at many genes concentrated in the distal PAS.^53^, ^156^ CFIm bound to UGUA at distal PAS will act as enhancer to recruit CPSF (via FIP1) and so stimulate 3′ end processing at a CFIm bound PAS.^52^, ^53^, ^8^, ^99^, ^100^ Experimental knockdown of CFIm, prevents recruitment of CPSF to distal PAS and so leads to proximal PAS usage and shorter 3′UTRs of about 800 genes within the human genome.^157^ For some mRNA, shortened 3′UTRs will lead to increased protein production, as has also been shown for some 3′UTRs shortened in glioblastoma cell lines that have naturally reduced CFIm.^157^, ^156^ In an auto-regulatory loop, CFIm68 provides itself with dosage control through two PASs in the 3′ UTR of its own mRNA; high cellular CFIm68 levels will recruit CPSF to the distal PAS and so generate a long 3′UTR with mRNA-destabilising elements that will gradually lower cellular CFIm68 levels. Once CFIm68 has dropped below a threshold the proximal PAS is now used and concordantly leads to higher CFIm68 expression.^101^

Protein-protein interactions of many SR proteins are controlled via phosphorylation and in the case of CFIm68/59, phosphorylation of its RS domain will prevent its interaction to FIP1 in CPSF.^53^ Consequently, hyperactivity of CLK2 kinase on CFIm68/59 as seen in several cancer cell lines prevents CFIm activity, as well as its nuclear local concentration into liquid–liquid phase separation foci – possibly by affecting its ability to interact with other SR proteins and CPSF.^158^, ^53^, ^100^

This inactivation of CFIm by hyperphosphorylation also explains how CFIm68 can be highly expressed in human hepatocellular carcinoma, breast cancer, and gastric cancer cells, but still be inactive and result in short 3′UTRs. These shorter 3′UTRs are corelated with higher proliferation, migration, and invasion of cancer cells.^159^, ^160^ CFIm may not be the only CPA factor that is regulated by phosphorylation, as APA can also be affected by the mammalian target of rapamycin (mTOR) pathway.^161^ mTOR will activate several kinases to regulate cell proliferation and its depletion is associated with several human diseases.^162^ Unscheduled activation of mTOR in mice leads to widespread 3′UTR shortening and overexpression of proteolytic enzymes which may contribute to general cellular metabolic disfunction.^161^

#### Variations of core CPA factors

*In vitro*, CFIm is not strictly required for the cleavage reaction and therefore can be classified as facultative enhancer of 3′ end processing.^43^, ^44^ Systematic depletion-studies identified PCF11 (CFII) and CstF64 as two core CPA factors that affect PAS-site usage and APA. As both factors are essential for the cleavage reaction, their depletion may generally result in less efficient 3′ end processing with concomitant transcription termination defects, as is seen for *pcf11*-hypomorph mutants in budding yeast.^84^, ^87^, ^3^, ^86^

PCF11, as part of CFII binds to G-rich sequences just adjacent to CstF some 30 nt downstream of the cleavage site and is required for efficient 3′ end processing at the first available PAS.^7^, ^102^, ^103^ Accordingly, high PCF11 levels in neuroblastomas, urothelial, breast, head, neck and prostate cancer result in shortened 3′UTRs.^163^ In contrast, low expression of PCF11, as seen in non-differentiated mouse myoblast cells, generally shifts PAS usage downstream.^7^ As detailed below, this provides a different intricate level of feed-back-control, as most PCF11-mRNA transcripts are usually prematurely terminated at an intronic PAS located in the first intron of PCF11. Low cellular PCF11 levels instead will allow the full-length mRNA and protein to be produced.^7^, ^103^

CstF subunits CstF64 and its paralogue CstF64τ bind to U/GU-rich sequences downstream of PAS to stimulate the cleavage reaction.^36^, ^37^ As classic, first described example of APA, CstF64-levels steadily increase during B cell activation and will shift PAS usage at the IgM locus from distal to proximal.^23^, ^61^ This results in expression of a shorter, non-membrane-bound form of IgM.^60^, ^61^ Such upregulation of CstF64 during higher proliferation rates, can also be seen in several tumour cell lines, including hepatocellular carcinoma, melanoma, prostate, breast cancer, pancreatic, liver cancer and renal cancer.^164^ In contrast, CstF64/64τ depletion results globally, just as PCF11 depletion, in usage of distal PAS.^19^, ^102^

Thus, depletion of PCF11 and/or CstF64/64τ, two essential CPA factors beautifully demonstrates the modality of the 3′ end processing reaction. As with PCF11 depletion, lower cellular CstF64 concentrations will delay deposition of a complete CPA complex on a PAS and cause transcriptional read-through to the next PAS. The transcripts that can be recovered in PCF11- or CstF64/64τ-depleted cells probably only represent a fraction of all transcripts, with many being non-functional and non-terminated at a PAS.^89^ As elongation speed drastically reduces downstream of the first PAS, it is possible that CPA complexes have more time to assemble at distal PASs, so favouring distal PAS usage in sub-optimal growth conditions.^95^

In contrast and as another consequence of this modal architecture of PASs, high cellular levels of CPA factors not only lead to 3′UTR shortening, but can also increase the use of intronic PAS.^66^, ^65^, ^69^ The resulting transcripts can still produce truncated proteins, but in several cases truncated proteins will interfere with the function of the full-length protein, as they only provide a fraction of its functionality.

#### Consequences of alternative polyadenylation

As 3′UTRs often contain miRNA and regulatory RBP binding sites, a change in 3′UTR length can modulate the mRNA’s stability, translatability and localisation.^165^, ^166^, ^167^, ^168^ Thus, the shorter 3′UTRs observed in highly proliferating cells will lack regulatory binding sites and allow such mRNA to escape post-transcriptional regulation.^169^, ^71^ As described above, in some cases shorter 3′UTRs will result in increased stability and higher protein production from a given mRNA. For example, a mutation in the Cyclin D1 encoding CCND1 gene that is associated with rare lymphomas, creates a new pre-mature PAS, which, if used, removes most of its 3′UTR containing destabilization elements normally present in the CCND1 mRNA. This truncated CCND1 mRNA has a much longer half-life than the full-length mRNA.^170^ General 3′UTR shortening has been observed in some cancers, including breast, lung and gastric cancers and in several cases been correlated with higher expression of some proliferation marker genes.^153^, ^156^, ^71^, ^155^ However, it is now clear that shorter 3′UTRs do not universally correlate with higher abundance and stability of the mRNA and so not necessarily will translate into higher protein production.^63^, ^171^, ^172^ Instead, it may be helpful to liken core CPA and accessory 3′ processing factors to transcription factors that act in a combinatorial manner on groups of genes that carry the same 3′ processing enhancer elements.^63^ To understand the complex effects that APA has on the expression of individual genes, we will undoubtedly have to combine scrutiny at single gene levels, with systematic approaches involving machine-learning and artificial intelligence-driven language transformers to build comprehensive models that encompass all stimulatory or inhibitory RBP-RNA interactions. For example, hnRNP family proteins, such as the polypyrimidine tract binding protein (PTB), can bind to CU-rich sequences downstream of the cleavage site and there compete with CstF binding.^173^ Similarly, SR proteins form intricate regulatory networks, such as SRSF3 which can recruit FIP1, but is counteracted by SRSF4.^174^ For a more extensive discussion of APA, the reader is referred to several comprehensive, recent reviews.^175^, ^176^, ^177^

#### Core CPA modulation by external agents

Above we considered the effect that pathological or natural variations to CPA factors may have. However, APA as an indication of transcription termination defects can also be caused by external stimuli. For example, cellular levels of the CPA endonuclease, CPSF73 are carefully maintained by controlled and balanced proteasome-mediated degradation.^21^ Lower levels of CPSF73, result in large transcription termination defects and interfere with proliferation of cells.^178^ In breast cancer cells this leads to interference with a cell’s ability to migrate and so provides a measure of how global 3′UTR changes can affect physiological traits.^21^ A cytokine production inhibitor JTE-607, also inhibits CPSF73. As a result, cellular treatment with JTE-607 leads to transcription termination defects and preferential distal PAS usage in a dosage dependent manner.^22^ Cell lines with high CPA activity are more susceptible to JTE-607 mediated inhibition, possibly, as CPSF73 is maintained at limiting concentration within CPA. As many cancer cells have a high CPA activity, JTE-607 bears hope as an adjuvant therapy specifically targeting highly proliferative cells.^22^

Cellular stress, such as osmotic or heat shock induce strong transcription termination defects that can profoundly affect the proteome of a cell.^179^, ^180^, ^181^ Stress related transcription termination defects appear to converge signalling cascades on CPA factors and so influence CPA deposition on PAS.^182^ We already mentioned that cancer associated hyperactivity of CLK2 results in hyperphosphorylation od CFIm68 and so inhibits its function.^53^ Paradoxically, CFIm appears to require some phosphorylation of its subunits, as treatment of nuclear extracts with phosphatases also inhibits the cleavage reaction.^183^

#### Pathogens affecting 3′ end processing

Several viruses have evolved effector molecules that interfere with 3′ end processing and consequently lead to large transcription termination defects.^184^, ^185^, ^186^, ^187^ As with core CPA factor mutations, the action of the viral effectors can be detected on the RNA level as global 3′UTR lengthening.^185^

A curious case is provided by ICP27, an immediate early gene encoded by HSV-1. Cellular HSV-1 infection induces wide-spread 3′ end processing and encompasses termination defects at many host cell genes, which correlates with reduced mRNA export and translation.^187^, ^188^ Inhibition of CPA function is partially mediated by ICP27 which binds to several components of CPSF (FIP1, CPSF160, CPSF73 and CPSF30) and results in deposition of a less active CPA complex on PAS-containing pre-mRNA.^186^ However, at the same time, ICP27 can also bind to GC-rich upstream sequence (UPS) elements that then act as 3′ end processing enhancers.^186^, ^189^ When located in host cell pre-mRNA, ICP27 binding to UPS will stimulate 3′ end processing and often result in distal to proximal APA, or even IPA. In combination with viral PAS, UPS is a pre-requisite to stimulate 3′ end processing.^186^ Understanding the different structural contacts between ICP27-CPA interactions in the repressive and the stimulating mode promises to provide mechanistic detail about CPA regulation and activation. Intriguingly, genes that show termination defects in HSV-1 also show reduced histone deposition, an effect that has been attributed to the ICP22 immediate early protein. This argues that chromatin modifications play a role in determining the site of transcription termination.^188^, ^190^

Influenza A virus (IAV) encodes the non-structural protein 1 (NS1), which is essential to counteract cellular host defence responses.^191^ NS1 broadly acts to inhibit signalling cascades that lead to the gene induction of immediate early and interferon stimulated genes. However, the NS1 of some IAV strains has also been shown to bind CPSF30 (aka CPSF4) in its RNA-recognition domain.^192^ CPSF30 encodes five Zn-fingers of which ZF2-ZF3 are required to contact AAUAAA of the central PAS.^33^ NS1 binding to ZF2-ZF3 of CPSF30 inhibits PAS recognition and so precludes CPA assembly on PASs.^184^ As a consequence of NS1 action, mRNA is not polyadenylated at the usual PAS resulting in transcripts that extend due to a termination-defective RNAPII.^181^, ^193^, ^194^ In some cases, alternative downstream PAS can be used to generate export competent mRNA.^185^ However globally, NS1-CPSF30 interaction results in large reductions of cellular proteins, especially of those whose mRNA are induced upon IAV-infection and so provides an efficient host-shut-off mechanism.^185^ It is therefore surprising that non-human adapted or zoonotic IAV strains show reduced affinity to CPSF30.^195^ In contrast, upon adaptation to the human population, NS1-CPSF30 interaction is positively selected for, and zoonotic or recently zoonotic IAV-strains gradually acquire mutations that support NS1 binding to CPSF30.^196^ Thus, in summary, the evolution of NS1 upon adaptation to the human host and the observation that a functional NS1-CPSF30 interaction mediates efficient host-shut-off, provides a striking example of how PAS selection and APA modulation can affect gene expression. As discussed below, some of these termination defects appear to involve drastic changes to histone modification and the three-dimensional architecture of chromatin, but the causal contribution of each of these factors is presently unclear.^188^, ^197^ As in budding yeast, chromatin compaction and modification appears to contribute significantly, if not epistatically, but in ill-defined ways to the ability of RNAPII to coordinate 3′ end processing and to be disengaged from the DNA template.^198^, ^199^, ^200^

### Scenario 2: Fusion transcripts (the more is not the merrier)

In healthy humans, stochastic read-through transcription extends on average 5 kb beyond a PAS and so in most cases ends before the start of the downstream gene.^144^ In contrast, in several cancer cell lines, read-through through of PAS is more frequent and can lead to 3 subtypes scenarios (a–c, see Figure 3) of bi-cistronic mRNA formation.^201^, ^202^, ^203^

#### Scenario 2a: Fusion transcripts

Splicing of the last intron depends on deposition of the CPA complex on the PAS.^204^ In the absence of CPA assembly, the last 3′ splice site (ss) of the upstream gene may not be recognised and instead the first 3′ ss of the downstream gene is used, resulting in a fused mRNA.^205^, ^144^, ^206^ If such chimeric mRNA is translated this may lead to gain or loss of function of one or both proteins and deregulation of their functions.^203^, ^207^ As with fusions created by DNA translocations, these aberrant proteins can have oncogenic potential. Thus, transcription induced protein fusions may provide a source of non-DNA encoded cancer heterogeneity.^208^, ^205^

In clear cell Renal Carcinoma cells (ccRCC) transcription read-through is in many cases associated with a mutation of the SETD2 histone (H3K36) methyltransferase and can extend into downstream genes.^205^ However, even though 20% of downstream genes show “read-in” stemming from upstream gene transcription termination defects in ccRCC cells, only 6% of the read-in significantly affects expression levels of the downstream gene.

One example for a fusion caused by splicing (scenario 2a), is the read-through chimaera ZNF577–ZNF649. ZNF577–ZNF649 consists of two mRNA encoding zinc-finger transcriptional regulators and has been associated with prostate cancer.^203^ Many other examples have been deduced from deep sequencing of prostate cancer cell lines of which 70% can be experimentally confirmed.^207^ A prerequisite for splicing induced fusions appears to be a relatively close distance of the 5′ ss of the upstream to the 3′ ss of the downstream gene with a median of 2 kb.

Finally, in some cases, read-through transcription can interfere with transcription of independent transcript units in cis – either by interfering with transcription from downstream promoters, or by provoking collisions of RNAPII molecules transcribing towards each other, which can result in the downregulation of one or both.^22^, ^15^, ^16^, ^209^, ^210^, ^211^

#### Scenario 2b: Posttranscriptional processing

A second variation of the scenario 2 is when the usage of the upstream PAS is very slow and so results in read-through transcription to the next available and possibly stronger PAS (Figure 3b). However, PAS cleavage will eventually occur and release an uncapped transcript.^212^, ^27^ Such uncapped transcripts would usually get degraded unless protected by a nuclease-resistant 5′UTR structure.^212^ Thus, in ccRCC cells, one gene into which read-through transcription extends, is the anti-apoptotic oncogene BCL2.^205^, ^213^ Following scenario 2a, read-through of the upstream KDSR PAS will generate a KDSR-BCL2 fusion transcript. Surprisingly, 3′ end formation at the KDSR PAS will eventually occur (scenario 2b) and so create an uncapped BCL2 mRNA.^212^ However, as the 5′UTR of BCL2 is highly structured and N6-methyladenosine (m6A)-modified, this uncapped BCL2 mRNA is stable and can, as with the regularly transcribed BCL2 mRNA, be translated. This results in net higher BCL2 levels, with overall tumour-supporting effect.^213^, ^202^

#### Scenario 2c: circRNA

In this scenario, spliced chimeras form circular (circ)RNA through back-splicing of a downstream 5′ss to an upstream 3′ss.^214^, ^215^ circRNA were originally discovered in plants, but their function, prevalence and biogenesis remained elusive for many years.^216^, ^42^, ^217^ It is now clear that circRNA are increasingly formed when RNA splicing or 3′ end processing factors are limiting.^218^ Studies in Drosophila and human cells revealed that depletions of transcription termination factors including CPSF73 (aka CPSF3), CPSF30 (aka CPSF4) and the exonuclease XRN2 increase the accumulation of circRNA.^218^ For example, the depletion of mRNA 3′ end processing endonuclease CPSF73 increased the formation of circRNA by 50-fold in an artificial reporter system, underscoring the importance of the termination machinery in preventing circRNA-formation.^218^ On the other hand, in biological situations, where 3′ end processing is impaired, as during cancerous transformation, transcriptional read-through can cause circRNA formation and result in the generation of scrambled transcripts that originally may have been misinterpreted as genomic rearrangements.^219^

While the formation of chimeric mRNA may directly affect the function of neighbouring genes, the accumulation of circular RNA (circRNA) have a direct effect on the global regulation of gene expression.^214^ CircRNA can act via multiple mechanisms, most commonly, the very stable circRNA can act as microRNA (miRNA) sponges, that sequester miRNAs and so affect the silencing of their true targets.^220^, ^215^ In addition, circRNA were also reported to interact with proteins^221^ and may be translated^222^ or regulate transcription.^223^ All these properties make circRNA potent gene expression regulators. Not surprisingly deregulation of their synthesis and accumulation is associated with cancer.^219^

As read-through transcription in cancer can span many kilobases, some circRNA can combine genes that are spaced far away from one another. For example, a circRNA formed by TTTY15 and USP9Y, which are separated by 9 kb, is overrepresented in prostate cancer tissue samples. Similarly, circRNA RB1-ITM2B originates from a read-through transcript of the tumour suppressor RB1 gene into the downstream ITM2B as detected in melanoma, lung and bladder cancers.^219^

### Scenario 3: Premature or promoter proximal termination (clear the clutter)

Genes that respond to extracellular signals and signalling cascades are often characterised by high promoter proximal accumulation of RNAPII.^150^, ^224^, ^225^ Although such accumulation was initially interpreted as stalled RNAPII molecules that were poised to enter transcription, more recent data suggests that these represent RNAPII populations that are turned over close to the promoter through a high rate of transcription termination.^224^, ^225^, ^226^, ^227^, ^228^, ^229^, ^230^, ^231^ Such promoter proximal transcription termination (PPT) is also referred to as RNAPII attenuation or premature transcription termination (PTT) and can be distinguished into PAS-mediated PPT and non-PAS PPT. Non-PAS PPT will generate non-polyadenylated transcripts, that are consequently nuclear, and unstable, and here degraded by nuclear RNA quality control mechanisms involving the nuclear RNA exosome.^232^, ^233^ It should be noted that at most promoters, initial transcriptional activity is non-directional and will generate antisense and sense transcripts.^234^, ^232^, ^235^, ^236^, ^237^, ^238^ Early termination and degradation of these divergent noncoding transcripts helps to direct most transcription into the coding direction and limit spurious transcriptional activity.^239^, ^240^, ^241^ Although we still lack a complete picture of the molecular machinery responsible for promoter proximal termination, it currently appears that different mechanisms lead to turnover in response to varied environmental conditions at different genes.^242^, ^243^, ^244^, ^230^, ^245^ These different pathways may respond to different signalling cascades and external stimuli and so shape a stimulus-specific transcriptional response.^149^, ^246^, ^245^

#### PAS-mediated PPT

Roughly 90% of all predicted PAS reside in introns and there could in principle lead to premature transcription termination or IPA.^59^ As discussed above, IPA is increased in certain cancer cell lines, where CPA factors are upregulated and here may contribute to their proliferation rate.^66^, ^65^ But in other tissues, such as neurons, IPA can be used to provide rapid control of gene expression. Mapping of PAS usage in human motor neurons revealed a high proportion of transcripts that terminate at PAS proximal to the 3′UTR.^247^ In motor neurons, this equilibrium is dependent on the CFII factor CLP1. A single point mutation pR140H, generates a dominant negative CLP1, which leads to globally decreased IPA and consequent transcription of more full-length genes, In the dish, pR140H results in pathological neuronal differentiation. Patients carrying pR140H, suffer from a disease called pontocerebellar hypoplasia type 10 (PCH10), which leads to microcephaly, motor-neuron disease, tonic-clonic seizures, as well as atrophies in the brainstem and cerebellum.^247^, ^248^ Experimental deletion of CLP1, leads to global increase in IPA, which results in equally abnormal neuronal differentiation and so indicates that premature transcription termination plays an important role in maintaining healthy neuronal cells.^247^

To reinforce the view that premature termination and consequent RNAPII recycling and RNA degradation is an important mechanisms to control gene expression, mutations in core exosome subunits, responsible for the turnover of prematurely terminated pre-mRNA, cause phenotypes very similar to PCH10.^249^ As described above, the second CFII subunit PCF11, surprisingly affects IPA in opposite ways to CLP1. Notably, PCF11 depletion leads to globally reduced IPA. Thus, PCF11-mediated premature termination over intronic PAS has been shown to regulate the expression of transcription factors, in particular 3′ end processing factors.^103^ Amongst those and as described earlier, PCF11 autoregulates its own expression through IPA.^103^, ^7^ Since PCF11 plays roles in regulation of premature transcription termination and APA at gene 3′ ends, its mutations are associated with cancer.^250^ However, it remains to be elucidated to what extent deregulation of PCF11-dependent attenuation contributes to human diseases. The opposing effects that mutation of these two CFII factors have, indicates that not only dysfunction of individual cleavage and polyadenylation factors but also the overall remodelling of interactions within the complex may contribute to the regulation of PAS selection.

However, IPA will only control a small number of genes, as IPA is normally suppressed by “telescripting”. As PAS resemble the U1 snRNP recognition sequence, U1 snRNP binding may occlude the intronic PAS and so prevent CPA binding.^251^, ^67^, ^68^, ^70^, ^233^ Conversely, at weak 5’ splice sites, U1 snRNPs assembly is stochastically less likely and CPA assembly and IPA favoured.^69^

#### Non-PAS-mediated PPT

In addition to PAS-dependent PPT, several non-PAS-dependent PPT mechanisms have evolved to maintain gene expression homeostasis and responsiveness. Still currently being elucidated, these pathways are also covered in the reviews by Estell and West, as well as Porrua-Fuerte and Libri in this issue.

##### Integrator

One of the non-PAS PPTs is mediated by Integrator is a large, >1 M Da complex, consisting of 14 subunits, of which INTS11 and INTS9 share sequence homology with the CPA endonuclease CPSF73 (CPSF3) and its binding partner CSPF100 (CPSF2), respectively.^252^, ^253^ Integrator was first identified as endonuclease, required for termination of non-polyadenylated RNAPII transcripts, including snRNA and enhancer RNAs (eRNAs).^252^, ^254^ However, subsequent studies showed that integrator is also responsible for the termination of a large proportion of nascent pre-mRNA at promoter-proximal pause sites.^97^, ^230^, ^245^ Since Integrator is involved in global regulation of gene expression, mutations or depletions of its subunits were reported to affect multiple cellular processes and various developmental stages in vertebrates including ciliogenesis, haemopoiesis, adipogenesis, dorsal–ventral patterning, neurodevelopment.^255^, ^256^, ^257^, ^258^, ^259^ For example, a short deletion in INTS8 affects the composition of the complex and results in neurodevelopmental diseases manifested by an array of defects including intellectual disability, epilepsy, cataracts and dysmorphic features with the patients.^260^, ^261^

The importance of PPT to fine-tune gene expression is exemplified by the finding that Integrator is a crucial transcriptional coactivator in MAPK-signalling, a pathway that is affected in >70% of cancers. MAPK-signalling-dependent gene activation is diminished upon INTS11 depletion leading to reduced cellular growth of cancer cell lines with activating mutations in BRAF and KRAS oncogenes.^262^ Moreover, INTS6 was identified as a tumor-suppressor protein involved in cell cycle regulation via the Wnt-signalling pathway.^263^ These two examples suggest that in addition to the APA-mechanisms described above, deregulated gene expression in cancer cells is partly mediated by a failure to prematurely terminate transcription close to promoters.

##### Restrictor

Another complex implicated in the promotion of proximal termination is called Restrictor and consists of a zinc finger RNA-binding protein ZC3H4, and the RNAPII CTD-interacting protein WDR82. Restrictor attenuates unproductive ncRNA transcription, including products of bidirectional transcription from mRNA promoters (PROMPTs) and eRNAs.^237^, ^264^, ^238^, ^265^, ^266^ and reviewed by Estell and West in this issue. Although only recently discovered and characterised, mutation of ZC3H4 has been associated with pulmonary fibrosis^267^ while WDR82-mutation has been indirectly linked to rare developmental disorders.^268^ Taking its roles in regulation of RNA synthesis from mRNA promoters and enhancers into account, it is likely that more restrictor muttations will be identified that affect human health.

Both, Integrator and Restrictor, cooperate with phosphatases. Integrator with the PP2A-holo-complex and ZC3H4 with PP1-PNUTS. It appears that the adaptor PP1, PNUTS, takes on similar functions as the phosphatase module in budding yeast.^238^, ^265^, ^269^, ^97^

##### SETX-helicase

A third, yet ill-defined mechanisms may involve the SETX helicase, which has been shown to restrict transcription of interferon stimulated genes close to promoters and requires the nuclear exosome.^242^, ^149^ The existence of such a PPT mechanism would have evolutionary precedence, as the budding yeast homologue of SETX, Sen1 is the enzymatic component of the only budding yeast ncRNA termination complex Nrd1-Nab3-Sen1 (NNS). NNS termination requires RNA recognition of Nrd1 and Nab3, in conjunction with Sen1 helical activity and the phosphatase module of CPF.^270^, ^271^, ^272^, ^273^, ^274^, ^116^, ^275^, ^119^, ^276^ Similar to the CPA endonuclease CPSF73, Sen1 appears to be kept in an autoinhibited state until it engages with appropriate protein partners and its substrate.^277^ Mutation of SETX causes ataxia-ocular apraxia Type II (AOAII), a disease that manifests in adolescence with gait problems and is caused by increased neurodegeneration in the cerebellum.^278^ So far, this phenotype has only been related to Sen1/SETXs function during transcription elongation to prevent RNAPII stalling upon RNA-DNA-hybrid formation.^279^, ^280^, ^281^, ^282^, ^283^ But given the newer insights into the importance of PPT, particularly in neurons, it could be that some of the phenotypes observed in the absence of SETX are of pleiotropic character caused by a general imbalance of PPT. In agreement with this, SETX has been shown to cooperate with the exonuclease required for RNAPII disengagement.^279^, ^284^, ^285^, ^149^

Overall, the mechanisms providing premature termination of RNAPII regulate the transcriptome and prevent uncontrolled and unwanted production of RNAs into both, sense and promoter-divergent antisense directions. It is yet to be elucidated how exactly these mechanism act in complex diseases characterised by global deregulation of transcription and processing of non-coding RNAs. For example, analysis of 251 post-mortem samples of brains tissues from 48 individuals with autism spectrum disorder revealed not only downregulation of neurodevelopmental protein-coding genes but also dysregulation in the synthesis of multiple primate-specific lncRNAs.^286^ Thus, much has yet to be learnt about how premature transcription termination adds an additional layer to the control RNA synthesis and to the maintenance of gene expression homeostasis in the healthy and diseased cell.

## Perspective

Transcription termination requires removal of nascent RNA from the transcription elongation complex (TEC). Primarily, this can be achieved by endonucleolytic cleavage in conjunction with an RNA exonuclease that upon degradation will pull the nascent RNA out of the elongating RNAPII complex. The two major endonucleases identified to date are contained within CPA and the Integrator complex, which both require phosphatases (PP2A and PP1/PNUTS) to complete transcription termination.^95^, ^269^, ^97^ In addition, a few mechanisms, involving Restrictor, SETX or specific T-rich DNA sequence (reviewed by Lopez Martinez and Svejstrup in this issue) may be able to destabilise transcribing RNAPII without transcript cleavage.

Gene expression homeostasis and fast adaptation to changing environmental stimuli require an agile and nimble system that can be changed, modulated and modified in an instant. The RNAPII termination mechanisms evolved in mammalian cells are clearly able to do just this. However, as emphasized in this review, this occurs at a cost of vulnerabilities that can affect both, the proteins and recognition sequences involved.

Our mechanistic understanding has greatly increased in the last five years and surely the future integration of genome wide sequencing data with AI-driven pattern recognition will greatly aid understanding the principles of the fine-tuning of individual proteins that promote or counteract RNAPII transcription termination.

## CRediT authorship contribution statement

**Pawel Grzechnik:** Writing – review & editing, Writing – original draft. **Hannah Mischo:** Writing – review & editing, Writing – original draft, Conceptualization.

## Declaration of competing interest

The authors declare that they have no known competing financial interests or personal relationships that could have appeared to influence the work reported in this paper.
